# Biliary Duct Granular Cell Tumor: A Rare But Surgically Curable Benign Tumor

**DOI:** 10.1155/1993/68037

**Published:** 1993

**Authors:** W. David Lewis, Joseph F. Buell, Roger L. Jenkins, Peter A. Burke

**Affiliations:** The Department of Hepatobiliary Surgery and Liver Transplantation Deaconess Hospital 110 Francis Street Boston MA 02215 USA

## Abstract

Granulosa cell tumors are rare benign tumors which may be found throughout the body. Rare cases are isolated within the biliary tree. If completely resected, surgical excision is curative.

A case of biliary duct granulosa cell tumor is presented with review of the world’s literature on this topic.


*HPB Surgery*, 1993, Vol. 6, pp. 311-317
Reprints available directly from the publisher
Photocopying permitted by license only

© 1993 Harwood Academic Publishers GmbH
Printed in the United States of America

### CASE REPORT

### BILIARY DUCT GRANULAR CELL TUMOR: A RARE BUT SURGICALLY CURABLE BENIGN TUMOR

W. DAVID LEWIS, JOSEPH F. BUELL, ROGER L. JENKINS and PETER A. BURKE

*The Department of Hepatobiliary Surgery and Liver Transplantation, Deaconess Hospital, 110 Francis Street, Boston, MA 02215, USA*

*(Received 10 February 1992)*

Granulosa cell tumors are rare benign tumors which may be found throughout the body. Rare cases are isolated within the biliary tree. If completely resected, surgical excision is curative.

A case of biliary duct granulosa cell tumor is presented with review of the world's literature on this topic.

KEY WORDS: Granular cell tumor, bile duct tumor

### INTRODUCTION

The etiology of biliary obstruction may occasionally be obscured by the presence of a benign tumor. Benign tumors of the extrahepatic biliary tract are uncommon, and in the USA the incidence is reported at 1 to 2 cases per 100,000 population[1]. These benign tumors consist primarily of hyperplastic (adenomatous and adenomyomatous), polypoid and granular cell tumors. Abrikossoff first described granular cell tumors (GCT) in 1926 and attributed their origin to striated muscle cells[2]. Granular cell tumors constitute only 1.1% of all benign tumors of the biliary tract and are considered rare in any location[3]. However, these nonmetastasizing lesions have been found in the oral cavity, skin or subcutaneous tissue, female reproductive tract[4], breast[5], pituitary gland[6], gastrointestinal tract[7], and bronchi[8].

Granular cell tumors occur in the biliary tree. They are most commonly seen in young black women, and generally arise at the confluence of the cystic, hepatic, and common bile duct leading to obstruction. The majority of patients present with abdominal pain and jaundice. A significant proportion however, present with painless jaundice or pruritus alone. Work up of obstructive lesions of the bile ducts generally includes radiologic visualization of the biliary system via percutaneous transhepatic cholangiogram (PTC) or endoscopic retrograde cholangiopancreaticogram (ERCP). In the case of GCT, a concentric smooth narrowing intrinsic to the bile duct is demonstrated. This finding is generally interpreted erroneously to represent a cholangiocarcinoma or primary sclerosing cholangitis. Misdiagnosis can lead to inadequate resection of a curable lesion, and result in treatment failure.

Forty-two cases of granular cell tumor of the biliary tree have been reported in the literature[9–41]. In this paper we report the forty-third case and only the third such case of a granular cell tumor isolated to the common hepatic duct.

### CASE REPORT

A twenty-seven year old white female was transferred from a Boston area hospital to the Hepatobiliary Service at the Deaconess Hospital for evaluation of bile peritonitis. The patient presented with nausea, vomiting, fever, diarrhea, abdominal pain and acholic stool. Her past medical history was unremarkable with the exception of a documented case of hepatitis A eleven months prior, with elevated bilirubin and alkaline phosphatase levels. Follow-up liver function testing revealed an elevated bilirubin of 2.5 mg/ml and an alkaline phosphatase of 2100 IU/L (nl <39 IU/L). A percutaneous liver biopsy demonstrated a nonspecific cholestatic picture with periportal fibrosis and bile duct proliferation. Post biopsy, the patient developed abdominal distension and signs of peritoneal irritation. A dimethyliminodiacetic acid (HIDA) scan demonstrated extrahepatic excretion of contrast and a subhepatic collection which proved to be bile by paracentesis.

On admission, she was mildly jaundiced without stigmata of chronic liver disease. She was afebrile. Her abdomen was distended with the liver edge 3 cm below the right costal margin. The spleen was 2 cm below the left costal margin. Abdominal exam elicited diffuse tenderness, rebound and shifting dullness. Laboratory findings included a normal prothrombin time but an elevated bilirubin of 2.9 mg/ml. The serum asparate aminotransferase was 69 U/ml (nl<40 U/ml); alkaline phosphatase 458 IU/L (nl<39 IU/L); and a white blood cell count of 12.600 per cubic millimeter. Paracentesis in the right anterior axillary line produced 2.5 liters of bile. Ultrasound and computerized tomography suggested a lesion in the upper biliary tract. ERCP was then performed, which revealed a concentric area of narrowing in the common hepatic duct (Figure 1). Dye was reflexed beyond the stratum and demonstrated the bile leak from the site of liver biopsy. During this procedure, a nasobiliary stent was placed in the common hepatic duct resulting in a fall in the bilirubin to 1.5 mg/ml; alkaline phosphatase to 279 IU/L and serum asparate aminotransferase to 33 U/ml.

At operation, a 2 cm smooth, oval tumor was found to involve the proximal common hepatic duct. The tumor had a firm, yellow, filamentous quality. Frozen section diagnosed the lesion as a granular cell tumor. Due to the lesion's proximal location, a Roux-en-Y hepaticojejunostomy was performed to assure adequate proximal resection margins. Distally, resection of the biliary system was extended to the level of the pancreas where frozen section confirmed the margins to be free of tumor. Permanent histologic examination of the specimen showed the resection margins to be free of tumor. The tumor was composed of large polygonal cells with distinct cell borders, small nuclei and an acidophilic granular cytoplasm. These granules were periodic acid-Schiff positive. Staining for S-100 protein was not performed.

The patient did well post operatively and was discharged home with normal liver functions six days post operatively. She remains in good health one year later.

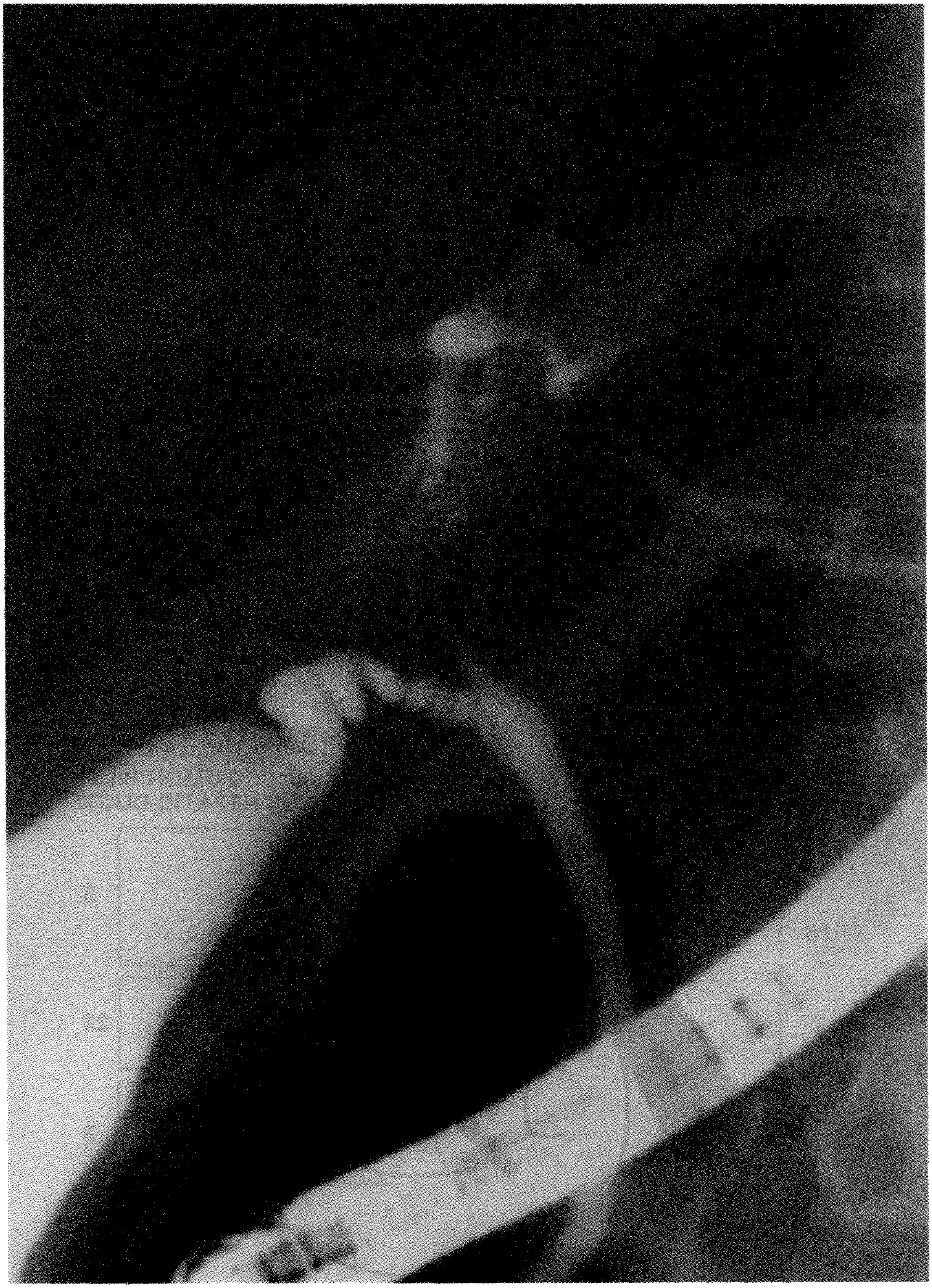

**Figure 1** ERCP of primary bile duct granulosa cell tumor. (*See colour plate at back of issue*).

### DISCUSSION

Granular cell tumors were first described by Abrikossoff in 1926 and Coggins reported the first case involving the biliary tree in 1952[2,9]. Since that time only forty-two cases of biliary involvement have been reported with only two isolated in the common hepatic duct[17,39]. In review, 95 percent of cases occurred in females[9–41], while 65 percent of all cases were in blacks. Two females were Asian, nine white, and four unspecified. The mean patient age was 33 (range 11 to 61). Duration of symptoms prior to the tissue diagnosis ranged from one week to six years.

Clinical symptoms at presentation are related to the anatomical location of the lesion. Plain (62%) and jaundice (36%) are the most commonly reported presenting symptoms. The vast majority of biliary GCTs involve the cystic, hepatic, and common bile duct (Figure 2). The only case of multifocality within the biliary tree involved the gallbladder, cystic duct, and common hepatic duct[32] (Table 1). Synchronous extra biliary lesions have also been described in six cases with five involving skin and a single lesion found in the stomach. Five cases have been reported to involve the pancreatic duct[9,30,42,43,44] While only a single case was found to involve the gallbladder alone[23]. Previously, two patients have had isolated involvement of the common hepatic duct[17,40]. Our case therefore is the third case of granular cell tumor isolated to the common hepatic duct in the literature. Fourteen of the 43 patients (35%) reported were worked up with transhepatic cholangiography or endoscopic retrograde cholangiopancreatography in which an area of circumferential narrowing in the biliary tree was demonstrated. Of note, correct diagnosis was not suspected in any of these cases preoperatively. Intraoperative

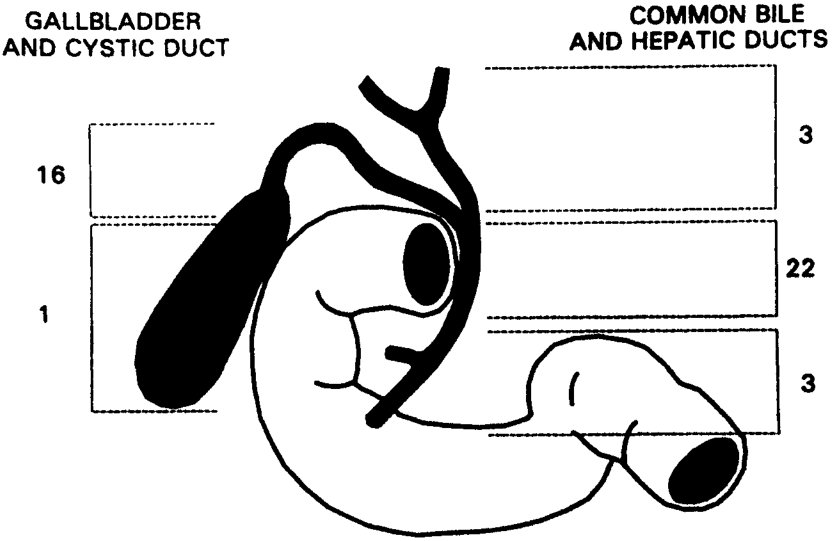

**Figure 2** Distribution of granulosa cell tumors within the biliary tree.

**Table 1** Multifocality of biliary duct granular cell tumors

| *Multifocality* | *Sex* | *Race* | *Location Biliary* | *Extra Biliary* |
|---|---|---|---|---|
| LiVolsi | Female | Black | CD | Skin |
| | Female | Black | CHD | Skin |
| Whitsnat | Male | Black | CBD | Skin |
| Assor | Female | Black | CBD | Skin |
| Aisner | Female | Black | CBD, CD, GB | |
| | Female | Black | CBD | Stomach |
| Orenstein | Male | Black | CD | Skin |

CBD = Common Bile Duck; CD = Cystic Duct; CHD = Common Hepatic Duct; GB = Gallbladder.

histological diagnosis is therefore the cornerstone of appropriate therapy. An important observation has been that the vast majority (41 of 43) of cases have been resected for cure. Despite the malignant nature observed in several granular cell tumors of the head, neck and most commonly the thigh, there have been no documented malignancies of the biliary tree[45,46]. Two cases of local recurrence have been documented but were felt to be due to inadequate resection margins[21,37] (Table 2). In the biliary tree, this diagnosis merely necessitates an adequate resection with clear margins for a cure. When a lesion is confined to the cystic duct alone, a simple cholecystectomy is the only procedure required.

The histogenesis of the granular cell tumor has been under controversy since Abrikossoff first attributed this lesion to striated muscle cells[2]. Others have postulated the cell of origin for this lesion to be fibroblasts[47], histiocytes[48], or mesenchymal storage cells[49]. In 1935, Feyter proposed that these lesions arose from a neural origin[50,51]. Later in 1949, Fust suggested that these tumors specifically arose from Schwann cells[52]. Evidence to support this theory came from the demonstration of the presence of S-100 protein within the tumors. S-100 protein has been found outside the central nervous system in the Schwann cells of the autonomic ganglia as well as in the granules of granular cell tumors. Despite persistent controversy, the Schwann cell theory of origin remains the predominant theory. In summary, granular tumors of the biliary tree are rare, benign tumors that most often occur in black females and present with pain or jaundice. Diagnostic procedures such as ERCP and PTC are sensitive in identifying lesions and their location but are nonspecific and unable to identify granular cell tumors. Due to confusion between diagnosis of granular cell tumors and cholangiocarcinoma it is essential to perform a frozen section and histologically confirm the

**Table 2** Complications associated with resection of biliary duct granular cell tumors

| *Author* | *Location* | *Procedure* | *Complication* |
|---|---|---|---|
| Dursi | CBD | Resection | Residual tumor |
| LiVolsi | CHD | Hepaticojejunostomy | Biliary fistula |
| Assor | CBD | Resection | Stenosis of CBD |
| | CBD | Resection | Biliary fistula |
| Orenstein | CBD | Incomplete resection | Residual tumor |

CBD = Common Bile Duct; CHD = Common Hepatic Duct diagnosis. It is also important to insure that surgical margins are free of disease to prevent local recurrence. Proper diagnosis and adequate surgical margins make the granular cell tumor of the biliary tree surgically, a curable lesion.

### *References*

1. Strong, E., McDivitt, R. and Brasfield, R. (1970) Granular cell myoblastoma. *Cancer*, **25**, 415–422
2. Abrikossoff, A. (1926) Uber myome ausgehend von der quergeslreiften villkurlichen muskulatur. *Virchows Archives*, **260**, 215–233
3. Christensen, A. and Ishak, K. (1970) Benign tumors and pseudotumors of the gallbladder. *Archives of Pathology*, **90**, 423–429
4. Herlig, A. and Gore, H. (1960) Tumors of the female sex organs. *Atlas of Tumor Pathology Series*, Washington DC Armed Forces Institute of Pathology. Part 2
5. Mulcare, R. (1969) Granular cell myoblastoma of the breast. *Annals of Surgery*, **98**, 662–667
6. Burslin, J., Spencer, H. and John, R. (1962) Myoblastoma of the neurohypophysis. *Journal of Pathology*, **83**, 455–461
7. Churg, J. and Work, J. (1959) Granular cell nodules of the gastrointestinal tract. *American Journal of Pathology*, **35**, 692–693
8. Rojer, C. (1965) Multiple endobronchial myoblastomas. *Archives of Otolaringology*, **82**, 652–655
9. Coggins, R.P. (1952) Granular cell myoblastoma of the common bile duct, report of a case with autopsy findings. *Archives of Pathology and Laboratory Medicine*, **54**, 398–402
10. Fialho, F. and Hilario, J. (1952) Rabdomioma granuloso do cristico. *Review of Brasilian Medicine*, **9**, 616–618
11. Duncan Jr., J.T. and Wilson, H. (1957) Benign tumor of the common bile duct. *Annals of Surgery*, **145**, 271–274
12. Serpe, S.J., Todd, D. and Baruch, H. (1960) Cholecystitis due to granular cell myoblastoma of the cystic duct. *American Journal of Digestive Disease*, **5**, 824–826
13. Goldman, L.I., Lemole, G. and Ellis, R. (1967) Granular cell myoblastoma of the cystic duct. *Journal of the American Medical Association*, **200**, 1185–1186
14. MacKay, B., Elliott, G.B. and Path, F.C. (1968) Granular cell myoblastoma of the cystic duct: Report of a case with electron microscopic observations. *Canadian Journal of Surgery*, **11**, 44–51
15. Whitmore, J.T., Whitley, H.P. and LaVerde, P. (1969) Granular cell myoblastoma of the common bile duct. *American Journal of Digestive Disease*, **14**, 516–520
16. Abt, A.B., Feinberg, E. and Kuntz, S. (1971) Granular cell myoblastoma of the extrahepatic biliary tract. *Mt. Sinai Journal of Medicine*, **38**, 457–461
17. Livolsi, V.A., Perzin, K.H. and Badder, E.M. (1973) Granular cell tumors of the biliary tract. *Archives of Pathology*, **95**, 13–17
18. Whisnant, Jr. J.D., Bennett, S.E. and Huffman, S.R. (1974) Common bile duct obstruction by granular cell tumor (Schwannoma). *American Journal of Digestive Disease*, **19**, 471–476
19. Reul Jr., G.J., Rubio, P.A. and Berkman, N.L. (1975) Granular cell myoblastoma of the cystic duct. A case associated with hydrops of the gallbladder. *American Journal of Surgery*, **129**, 583–584
20. Kittredge, R.D. and Baer, J.W. (1975) Percutaneous transhepatic cholangiography, problems in interpretation. *American Journal of Radiology*, **125**, 35–46
21. Dursi, J.F., Hirschl, S. and Gomez, R. (1975) Granular cell myoblastoma of the common bile duct: Report of a case and review of the literature. *Review of Surgery*, **32**, 305–310
22. Savage, A. and Devitt, P. (1977) Granular cell myoblastoma of the biliary tree. *Postgraduate Medical Journal*, **53**, 747–747
23. Ishii, T. and Iri, H. Yamamoto (1977) Granular cell myoblastoma of the gallbladder. *American Journal of Gastroenterology*, **68**, 38–44
24. Zvargulis, J.E., Keating, J.P. and Askin, F.B. (1978) Granular cell myoblastoma. A case of biliary obstruction. *American Journal of Diseases of Childhood*, **132**, 68–70
25. Farris, K.B. and Faust, B.F. (1979) Granular cell tumor of the biliary ducts. *Archives of Pathology and Laboratory Medicine*, **103**, 510–512
26. Jain, K.M., Hastings, O.M. and Rickert, R.R. (1979) Granular cell tumor of the common bile duct: Case report and review of the literature. *American Journal of Gastroenterology*, **71**, 401–407
27. Assor, D. (1979) Granular cell myoblastoma involving the common bile duct. *American Journal of Surgery*, **137**, 673–675

28. Bouquet, L., Visseizaine, C. and Grossin, M. (1980) Tumeur a celleles granuleuses des voies biliares. *Archives of Analytic Cytology and Pathology*, **26**, 360–364
29. Dewar, J., Dooley, J.S. and Lindsay, I. (1981) Granular cell myoblastoma of the common bile duct treated by biliary drainage and surgery. *Gut*, **22**, 70–76
30. Manstein, M.E., McBreatry, F.X. and Pellechia, P.E. (1981) Granular cell tumor of the common bile duct. *Digestive Disease Sciences*, **26**, 938–942
31. Mauro, M.A. and Jaques, P.F. (1981) Granular cell tumors of the esophagus and common bile duct. *Journal of Canadian Association of Radiology*, **32**, 254–256
32. Aisner, S.C., Khaneja, S. and Ramirez, O. (1982) Multiple granular cell tumors of the gallbladder and biliary tree. *Archives of Pathology and Laboratory Medicine*, **106**, 470–471
33. Penalba, C., Vallot, T. and Cerf, M. (1982) Les tumeurs a cellules granuleuses des voies biliares. *Annel Chir*, **36**, 723–726
34. Balart, L.A., Hines Jr., C. and Mitchell, W. (1983) Granular cell Schwannoma of the extrahepatic biliary system. *American Journal of Gastroenterology*, **78**, 297–300
35. Barber, C.J. (1984) Granular cell tumor of the cystic duct: A case of cholecystitis. *Journal Review at the College of Surgeons Edinburgh*, **29**, 56–57
36. Chandrasoma, P. and Fitzgibbons, P. (1984) Granular cell myoblastoma of the intrapancreatic common bile duct. *Cancer*, **53**, 2178–2182
37. Orenstein, H.H., Brenner, L.H. and Nay, H.R. (1984) Granular cell myoblastoma of the extrahepatic biliary system. *American Journal of Surgery*, **147**, 827–831
38. Yamashina, M. and Stemmermann, G.N. (1984) Granular cell tumor: Unusual case of mucocele of gallbladder. *American Journal of Gastroenterology*, **79**, 701–703
39. Cheslyn-Curtis, S., Russell, R.C.G. and Rode, J. (1986) Granular cell tumor of the common bile duct. *Postgraduate Medicine Journal*, **62**, 961–963
40. Butterly, L., Schapior, R. and LaMuraglia, G. (1988) Biliary granular cell tumor: A little known curable bile duct neoplasm of the young people. *Surgery*, **103**, 328–334
41. Timberlake, G. and Tachovsky, T. (1988) Granular cell myoblastoma of the cystic duct. *Military Medicine*, **15**, 398–399
42. Wellmann, K.F., Tsai, C.Y. and Reyes, F.B. (1975) Granular cell myoblastoma in the pancreas. *New York State Journal of Medicine*, 1270
43. Seidler, A., Burnstein, S. and Driveiga, W. (1986) Granular cell tumor of the pancreas. *Journal of Clinical Gastroenterology*, **8**, 207–209
44. Sekas, G., Talamo, T. and Julian, T. (1988) Obstruction of the pancreatic duct by a granular cell tumor. *Digestive Diseases and Sciences*, **33**, 1334–1337
45. Klima, M. and Peters, J. (1987) Malignant granular cell tumor. *Archives of Pathology and Laboratory Medicine*, **111**, 1070–1073
46. Cadotte, M. (1974) Malignant granular cell myoblastoma. *Cancer*, **33**, 1417–1422
46. Pearse, A.G.E. (1950) The histogenesis of the granular cell myoblastoma. *Journal of Pathology and Bacteriology*, **52**, 351–362
48. Vance III S.F. and Hudson, Jr. R.P. (1969) Granular cell myoblastoma. *American Journal of Clinical Pathology*, **52**, 208–211
49. Moscovic, E.A. and Azar, H.A. (1967) Multiple granular cell tumors. *Cancer*, **20**, 2032–2047
50. Feyter, F. (1935) Uber eine eigenartige geschwulstform des nervengerveles in menschlichen verdaunesschlauch. *Virchows Archives*, **295**, 480–501
51. Stefanson, K. and Wollmann, R. (1982) S-100 protein in granular cell tumors. *Cancer*, **49**, 1834–1838
52. Fust, J.A. and Custer, R.P. (1949) On the neurogenesis of so called granular cell myoblastoma. *American Journal of Clinical Pathology*, **19**, 522–535

(*Accepted 21 January 1990 by John L. Cameron*)

